# Erectile Dysfunction and Oxidative Stress: A Narrative Review

**DOI:** 10.3390/ijms26073073

**Published:** 2025-03-27

**Authors:** Dake Zhu, Quan Minh Pham, Chunlin Wang, Elena Colonnello, Dimitri Yannas, Bac Hoai Nguyen, Yan Zhang, Emmanuele A. Jannini, Andrea Sansone

**Affiliations:** 1 Chair of Endocrinology and Medical Sexology (ENDOSEX), Department of Systems Medicine, University of Rome Tor Vergata, 00133 Rome, Italy; gagakd35uk@gmail.com (D.Z.); quanpham.fsh@gmail.com (Q.M.P.); elena_colonnello@hotmail.it (E.C.);; 2 Department of Andrology and Sexual Medicine, Hanoi Medical University Hospital, Hanoi 100000, Vietnam; 3 Department of Infertility and Sexual Medicine, The Third Affiliated Hospital of Sun Yat-sen University, Guangzhou 510630, China; 4 Department of Experimental Medicine, Sapienza University of Rome, 00185 Rome, Italy; 5 Surgery Faculty, Hanoi Medical University, Hanoi 100000, Vietnam

**Keywords:** erectile dysfunction, sexual dysfunction, oxidative stress, reactive oxygen species, nitric oxide, endothelial dysfunction, smoking, male infertility, premature ejaculation, depression

## Abstract

Erectile dysfunction (ED) is a prevalent condition affecting male sexual health, characterized by the inability to achieve or maintain satisfactory erections. ED has a multifactorial pathogenesis in which psychological, hormonal, neurologic, cardiovascular, and lifestyle factors all contribute to a progressive decline of erectile function. A critical underlying mechanism involves oxidative stress (OS), an imbalance between reactive oxygen species (ROS) production and antioxidant defenses, which disrupts endothelial function, reduces nitric oxide (NO) bioavailability, and contributes to vascular dysfunction. This narrative review explores the interplay between OS and ED, focusing on the roles of ROS sources such as NADPH oxidase, xanthine oxidase, uncoupled nitric oxide synthase, and mitochondrial dysfunction. It examines the impact of OS on chronic conditions like hypertension, diabetes mellitus, hyperlipidemia, hypogonadism, and lifestyle factors like smoking and obesity, which exacerbate ED through endothelial and systemic effects. Emerging research underscores the potential of antioxidant therapies and lifestyle interventions to restore redox balance, improve endothelial function, and mitigate ED’s progression. This review also highlights gaps in understanding the molecular pathways linking ROS to ED, emphasizing the need for further research to develop targeted therapeutic strategies.

## 1. Introduction

Erectile dysfunction (ED) is the inability to achieve or maintain an erection sufficient for satisfactory sexual performance [1]. ED is one of the most prevalent male sexual dysfunctions [2,3], with a multifactorial pathogenesis that features both organic and non-organic risk factors [4,5]. Indeed, psychological (e.g., anxiety, depression, and stress [6]), hormonal (e.g., hypogonadism, hypothyroidism, hyperprolactinemia [7]), cardiovascular (e.g., hypertension, atherosclerosis, heart failure [8]), metabolic (e.g., obesity, metabolic syndrome, diabetes, dyslipidemia, gout [9,10]), neurologic (e.g., central and peripheral neuropathies [11,12,13]) risk factors can promote the development of ED [14], or its progression from subclinical to more severe forms [15]. Also, ED may be induced by pelvic surgery, medications (i.e., antidepressants, antihypertensives, antihistamines [16,17,18,19,20]), or lifestyle factors (i.e., smoking, substance abuse, alcohol, physical inactivity [21,22,23]). In a view based on systems sexology [24], this has proven that ED can be an early sign of more severe, underlying conditions, such as cardiovascular diseases (CVDs) and other non-communicable chronic diseases (NCDs), which share the same risk factors [5,25]. Other sexual dysfunctions, such as premature ejaculation [26], loss of control of erection and ejaculation (LCEE) [27]), performance anxiety, or “lost penis syndrome” (i.e., reduced penile sensitivity due to idiosyncratic masturbatory style) [28,29] can also lead to the development or progression of ED.

The physiology of erections is a complex interplay of penile anatomical compartments, vessels, and nerves [30]. The corpora cavernosa of the penis are two cylinders of smooth muscle surrounded by the tunica albuginea, functioning as erection chambers [31,32]. The blood supply comes from the internal pudendal artery, which separates into 3–4 branches into the penis [32]. Directly below the tunica albuginea is the subtunical venous plexus, which drains into emissary veins and eventually forms the dorsal veins of the penis [32]. The autonomic nervous system plays an integral role, with parasympathetic nerves involved in tumescence and the sympathetic nerves controlling detumescence. Due to the mechanism of erection being closely linked with the redistribution of blood in the penis, factors affecting the integrity of the vascular endothelium per se can impact erection.

**Figure 1:**
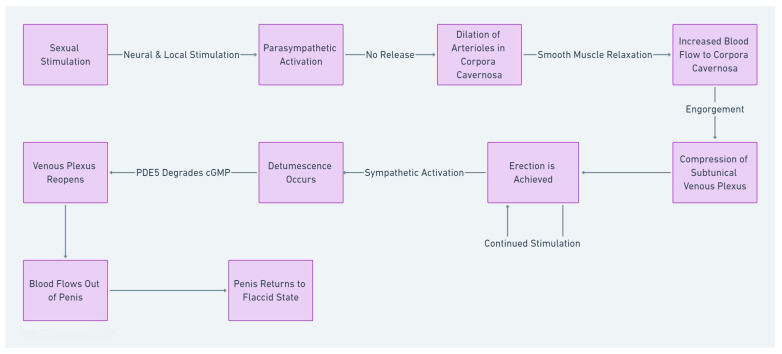
The mechanism of penile erection and detumescence.

Briefly, erection occurs following the transmission of sexual stimuli from the brain cortex to the spinal cord and subsequently to the sympathetic ganglia, partly mediated by testosterone [33,34,35]. At a molecular level, this results in the release of neurotransmitters, particularly nitric oxide (NO): NO is synthesized by the NO synthase enzyme (NOS), and particularly by two isoforms of this enzyme present in nerve terminations (neuronal NOS; nNOS) and in endothelial cells (endothelial NOS; eNOS) [36]. NO activates the soluble guanylate cyclase, subsequently catalyzing the reaction producing cyclic GMP (cGMP) from GTP; cGMP acts downstream, activating a kinase-mediated pathway which, ultimately, results in the relaxation of vascular smooth muscle and, therefore, in vasodilation. The corpora cavernosa thus becomes engorged with blood, compressing the subtunical venous plexus and effectively trapping blood within the penis. The breakdown of this mechanism is produced by the erectolitic enzyme type 5 phosphodiesterase (PDE5), which brings the intracellular cGMP concentration to the basal levels [37,38]. While traditionally, studies have concentrated on assessing PDE5 expression within several human tissues, very recently, it has been demonstrated that it is possible to dose PDE5 not only in the penis but also in the peripheral blood [39]. Detumescence occurs when the arterioles contract, allowing the venous plexus to reopen and enabling blood to flow out of the penis (Figure 1).

Oxidative stress (OS) has been shown to be detrimental to endothelial functions [40]. It is caused by an imbalance between the production and degradation of reactive oxygen species (ROS) in cells and tissues and the ability to detoxify these reactive products [41]. ROS are generated as by-products of oxygen metabolism [42]. At the low to moderate level, they act as essential signaling molecules [43]. However, under the effects of environmental stressors (i.e., UV, ionizing radiations, heavy metals, and pollutants) and xenobiotics (i.e., antiblastic drugs), a large amount of ROS is produced, surpassing the body’s ability to cope, thereby disrupting the balance of oxidant levels in the body, leading to OS [41]. OS has the ability to destroy different molecules and cellular structures, changing the normal function of organs and systems [44]. OS disrupts endothelial function, diminishes NO bioavailability, and contributes to vascular dysfunction, all of which serve as critical mechanisms underlying the onset and progression of ED [40].

## 2. Endothelial Function and ED

**Figure 2:**
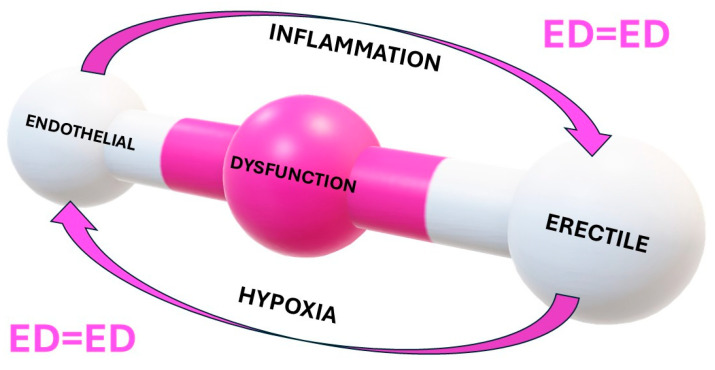
The biunivocal relationship between the first ED (endothelial dysfunction) and the second ED (erectile dysfunction) is supported by the two shared mechanisms of action, i.e., inflammation and hypoxia. In fact, chronic inflammation, like all non-communicable chronic diseases, produces chronic damage to the ability of the endothelium to activate the biochemical and mechanical machinery of erection. At the same time, the lack of erections, as in erectile dysfunction, reduces the tension of oxygen of the endothelial cells, thus exacerbating the proinflammatory action.

The endothelium (the inner lining of blood vessels) plays a crucial role in erectile function by producing NO, which, as stated above, is a key molecule for vasodilation and, therefore, erection [45]. Endothelial dysfunction, often caused by conditions like chronic low-grade inflammation, atherosclerosis, hypertension, and diabetes, impairs NO production, thus contributing to impaired smooth muscle relaxation and ED. This effect has been efficaciously popularized in the equation ED = ED, representing the common pathogenetic effect of the first ED (endothelial dysfunction) and of the second ED (erectile dysfunction) and the biunivocal relationship between the two EDs, being the first causative of the second, and vice-versa [46,47] (Figure 2).

Hence, it should not surprise that ED is considered an extremely reliable marker of systemic endothelial dysfunction [47] and a potential early indicator of CVD [48]. In fact, according to the “artery size hypothesis”, since penile vessels feature a smaller caliber than coronary arteries [49], symptoms of ED may precede those of CVD by several years [47,50]. In vascular-related ED, elevated OS leads to impaired endothelial function by reducing NO availability and increasing vasoconstriction [40,51]. In fact, when the production of ROS exceeds the body’s antioxidant scavenging abilities, they react with NO, leading to the production of reactive nitrogen species (RNS), such as peroxynitrous acid (ONOOH) and peroxynitrite (ONOO–). Not only does this result in reduced availability of NO for vasodilation, but ROS and RNS can also induce the proliferation of smooth muscle cells and stimulate the expression of cell adhesion molecules, mostly ICAM-1 and VCAM-1, that control the inflammation on the vascular level by leukocyte migration and adhesion [52]. Endothelial dysfunction typically represents an early stage of vascular injury, which can advance to more serious conditions such as atherosclerosis in the systemic circulation [40]. Recent evidence has shown that the immune response initially occurring in endothelial dysfunction is indeed the grounds upon which progressive atherosclerotic damage occurs [53].

## 3. Reactive Oxygen Species

ROS (and, to an extent, RNS) are byproducts of oxygen metabolism, resulting from incomplete reduction in molecular oxygen in the course of aerobic cellular processes (Table 1). ROS include free radicals like superoxide anions, hydroxyl, peroxyl, and hydroperoxyl radicals, as well as nonradical species like hydrogen peroxide and other peroxides [54].

These molecules are part of the pathophysiology of several conditions due to their role in inflammation and are prominently featured in the “free radical theory of aging” [55,56], which postulates that progressive damage resulting from OS (regardless of its source) could be the main driver for the plethora of aging-related diseases and conditions. It is important to note that this is only applicable when there is an excess of reactive oxygen species (ROS) in the body. While still a topic of debate, it is believed that ROS may play a crucial role in ATP formation through direct electron transfer and catalysis beyond the active sites of enzymes, as suggested by the murburn concept [57,58]. ROS are also part of many physiological processes involved in immune response, as well as essential signaling molecules and second messengers [59]: as such, it could be argued that the reduction in the host’s scavenging capacities, rather than the increase in ROS, is the primary pathogenetic mechanism.

### 3.1. Sources of ROS

#### 3.1.1. Endogenous Sources of ROS

The mitochondria and NADPH oxidases are among the primary intracellular sources of ROS [60]. Other sources of ROS include xanthine oxidase, uncoupled NO synthase, and others [61]. It is worth mentioning that these sources can influence each other, with the activity of one amplifying the function of the others, resulting in a progressive increase in OS [62].

**Mitochondria:** The mitochondrion, well-known as “the powerhouse of the cell” [63], heavily relies on oxidative phosphorylation to break nutrients down into energy [64,65]; this process produces much more ATP than would be produced by aerobic glycolysis [64], but at the same time is a major source of ROS [66]. According to the chemiosmotic rotary ATP synthesis model, mitochondria generate ROS as a result of electron leakage, which occurs as a byproduct of normal oxidative phosphorylation and ATP production [66]. During oxidative phosphorylation, the mitochondrial electron transport chain can “leak” electrons, leading to the formation of superoxide. Recently, in line with the new murburn concept, reactive oxygen species (ROS) generated during redox metabolism directly participate in reactions that synthesize ATP without the need for a proton gradient [57]. However, if the dynamic balance between ROS and antioxidants is disrupted, severe consequences may occur. ROS overproduction by mitochondria can lead to oxidative damage to mitochondrial proteins, membranes, and DNA, impairing the ability of mitochondria to synthesize ATP and to carry out their wide range of metabolic functions, including the tricarboxylic acid cycle, fatty acid oxidation, the urea cycle, amino acid metabolism, and heme synthesis [67]. In penile tissue, mitochondrial dysfunction might potentially elevate ROS levels, contributing to endothelial dysfunction, which, in turn, reduces NO availability. Although the role of mitochondrial superoxide in erectile dysfunction has not been thoroughly investigated, a potential vasoconstrictive ability has been hypothesized [68], which might contribute to understanding the pathophysiology and be applied in managing ED.

**NADPH Oxidase**: NADPH oxidases (NOX) serve as a regulated source of ROS production [69]. They are a group of transmembrane enzymes whose role is to facilitate the transfer of electrons from cytosolic NADPH to molecular oxygen, resulting in the formation of superoxide anions [70]. ROS generated by NOX is vital for specific host defense mechanisms, such as neutrophil-mediated pathogen clearance [69]. However, excessive ROS production from these oxidases can induce OS, resulting in tissue damage and contributing to inflammatory diseases (such as rheumatoid arthritis) [71]. Furthermore, these ROS are associated with aging and a variety of disorders, including hypertension, diabetes mellitus, hypercholesterolemia, and sickle cell disease [72,73,74].

**Xanthine Oxidase**: Xanthine oxidase (XO) is a multifaceted enzyme that catalyzes the catabolism of hypoxanthine into xanthine, followed by its conversion into uric acid. During this reaction, it produces ROS, such as hydrogen peroxide and various superoxide radicals [75,76]. Under physiological conditions at low concentrations, these substances can act as non-enzyme electron carriers involved in energy supply [57]. Elevated serum cholesterol, liver damage, systemic inflammation, and hypoxic conditions can increase XO secretion via hepatic and other visceral pathways into the bloodstream and bind to the endothelial cell surface, initiating the overproduction of superoxide [77]. The role of ROS generated by XO in the penis remains unclear, requiring further research to determine its potential involvement in ED.

**Uncoupled Nitric Oxide Synthase**: There are three isoforms of NOS: nNOS and eNOS are constitutively expressed, whereas the third one, inducible NOS (iNOS), is expressed in response to cytokines [78]. In physiologic conditions, NOS isoforms generate NO when an adequate cofactor (e.g., tetrahydrobiopterin, BH4) is available; however, when NOS becomes “uncoupled” due to reduced cofactor availability, superoxide is produced instead of NO. NOS uncoupling leads to decreased NO production while increasing the enzyme’s capacity to generate ROS [79]. OS induces superoxide generation mediated by XO, which leads to tetrahydrobiopterin depletion and S-glutathionylation, ultimately causing eNOS uncoupling [80]. Recent studies have revealed that eNOS uncoupling in the penis significantly contributes to the development of ED and localized OS [81].

#### 3.1.2. Exogenous Sources of ROS

There are several factors that serve as exogenous sources of ROS. These include nutrients, smoking, alcohol, drugs (such as halothane, doxorubicin, and metronidazole), industrial solvents, heavy metals (e.g., Fe, Cu, Co, and Cr), transition metals (e.g., Cd, Hg, Pb, and As), air pollution, physical stressors (like UV and X-rays), and lifestyle choices [82]. These factors trigger the extensive production of ROS, thus potentially leading to OS that directly impacts human health. While the role of endogenous ROS as hormetic agents is emphasized in the above discussion, the same conclusion cannot be confidentially withdrawn in case ROS originated from exogenous sources, which might be the field of interest for further research.

#### 3.1.3. Degenerated Products of Antioxidant Defenses

ROS are generated by all living organisms. Although organisms possess antioxidant defenses, such as enzymes, proteins, and vitamins, excessive ROS production or a malfunction in the antioxidant system can result in their accumulation, ultimately leading to a state of OS [83]. Several mechanisms, detailed below, could contribute to this imbalance between ROS formation and scavenging.

**Antioxidant Enzymes Becoming Dysfunctional:** Antioxidant enzymes like superoxide dismutase (SOD), catalase, and glutathione (GSH) peroxidase neutralize ROS under normal conditions. However, if these enzymes are damaged or depleted (e.g., due to OS, mutations, or degradation), their improper functioning can inadvertently lead to increased ROS production [84].

**Autoxidation of Antioxidant Molecules**: Some small-molecule antioxidants, like ascorbic acid (vitamin C) and reduced GSH, can undergo autoxidation when in excess or in the presence of transition metals like iron or copper. This reaction can paradoxically generate ROS [85].

**Degradation Products of Antioxidants:** Antioxidant compounds like polyphenols, carotenoids, and flavonoids can degrade into byproducts under extreme oxidative conditions or due to prolonged exposure to heat or light. Some of these degradation products may act as pro-oxidants, promoting the formation of ROS instead of quenching them [86].

**Imbalanced Antioxidant Defenses**: An imbalance between antioxidant defenses and ROS production can lead to an “antioxidant paradox.” For instance, high concentrations of antioxidant molecules can react with ROS in non-ideal ways, resulting in unstable intermediates that can further generate ROS [87].

### 3.2. ROS Scavengers and Antioxidants

The overproduction of ROS has been clearly linked to endothelial dysfunction, which is recognized as a potential cause of impaired erectile function [47]. Scavenging mechanisms play a critical role in mitigating this damage. Antioxidants target ROS to restore NO availability and enhance endothelial health [88].

**Enzymatic Antioxidants:** SOD, catalase, and glutathione peroxidase detoxify ROS, preventing cellular injury [89,90]. Their discovery underscored the importance of enzymatic regulation in scavenging ROS.

**Non-Enzymatic Antioxidants:** Compounds like vitamin C, glutathione, and α-tocopherol (vitamin E) neutralize free radicals. For example, α-tocopherol, a lipid-soluble antioxidant, halts oxidative chain reactions [91], while ascorbate (vitamin C) scavenges ROS at lipid–water interfaces [92].

Balancing the redox equilibria is important in maintaining body function and reducing damage from OS. However, the beneficial effects of using antioxidants are still a field of controversy that requires more evidence in the future. Under the perspective of the new murburn concept on the critical roles of ROS in bioenergetic systems [57,58], the overuse of antioxidants may disrupt redox homeostasis. As hormetic agents, physiological ranges or “beneficial levels” of ROS should be established to avoid the “antioxidant paradox” and ensure balance in ROS production and scavenger.

### 3.3. Chemical Reactivity of ROS

As already mentioned, ROS are essential signaling molecules involved in the development and progression of inflammation and inflammatory disorders. Increased ROS production by polymorphonuclear neutrophils at inflammatory sites leads to endothelial dysfunction and tissue damage [93]. Additionally, ROS act as key signals for inflammasome activation via mitogen-activated protein kinases (MAPK) and extracellular signal-regulated kinases 1 and 2 (ERK1/2) [94]. The abnormal regulation of inflammasomes is among the most important contributors to the exaggerated inflammatory response to exogenous pathogens and stressors [95].

ROS are highly reactive, to the point that, if left unaddressed, they can cause damage to DNA, proteins, and lipids, leading to cell death and aging symptoms. As stated elsewhere, this is prominently featured in the free radical theory of aging, introduced by Harman in 1954 and later revised as the mitochondrial free radical theory of aging, which links OS and mitochondrial dysfunction to aging-related changes [56]. However, this concept has undergone several revisions over the last decades. While the role of ROS in aging is more than plausible, some findings suggest that moderate ROS levels activate protective mechanisms, potentially extending lifespan—a concept termed mitohormesis [96,97]. While this can seem paradoxical, there are several examples in human metabolism showing that suppressing ROS production can have negative effects, such as acrosome reaction in spermatogenesis; therefore, a balance between ROS production and scavenging systems is necessary to preserve homeostasis [98]. Caloric restriction and endurance training, enhancing mitochondrial function, are promising strategies for mitigating aging effects [99,100,101].

## 4. Environmental and Lifestyle Contributions

Environmental toxins (e.g., air pollution, pesticides, heavy metals) and poor lifestyle choices (smoking, sedentary habits, high-fat diets) exacerbate OS, linking it to obesity, metabolic syndrome, and diabetes.

### 4.1. Psychological Distress and OS

Mental health issues, particularly stress-related disorders and psychological distress, negatively impact sexual performance and erection, and vice versa [102,103]. This connection thrives and endures through a multitude of pathways, both direct and indirect [102]. Interestingly, both psychological problems and sexual dysfunction are potentially related to OS, making it a key factor in worsening their impact on men’s health and opening new clinical implications of using antioxidants as adjuvant treatments [104,105,106].

Humans are continuously exposed to stressors from exogenous sources. Accordingly, all stressors provoke similar non-specific neuroendocrine responses, potentially breaking down the physiological defense against stress under prolonged exposure [104,107]. Indeed, sufficient physiological stress at low to moderate doses helps maintain a healthy oxidative milieu, while high-chronic supraphysiological stress doses increase oxidative damage [104,108]. OS is a primary cause of neurodegeneration; its involvement in the pathogenesis of major depressive disorder (MDD) is unequivocally established. OS and proinflammatory signaling have emerged as mainstays in the pathogenesis of MDD [109]. With the reveal of the OS-induced mechanisms in psychiatric disorders and neurological deficits [86,104,106], bidirectional interactions between stressors, especially psychological distress, and OS become more apparent [104].

Psychological stress activates the sympathetic nervous system (SNS) and the hypothalamic–pituitary–adrenal axis [104,110]. SNS releases catecholamines, including epinephrine and norepinephrine, which are needed for vital responses but accidentally upregulate the production of proinflammatory cytokines, such as IL-1, IL-6, and TNF [110]. On the other hand, the adrenal cortex synthesizes and releases cortisol, which increases gluconeogenesis and insulin resistance to ensure energy production and also plays an essential role in modulating inflammation during the acute phase [104,110]. When exposed to chronic stress, the overproduction of cortisol leads to glucocorticoid desensitization and resistance in the central nervous system, subsequently eliminating the anti-inflammation effect and triggering the accumulation of proinflammatories in the brain, elevating OS level [104,110]. By that mechanism, psychological distress significantly contributes to dysregulation redox homeostasis. Furthermore, psychological distress even affects the brain more severely through changing lifestyle factors, including eating disorders and low levels of physical activity, thus magnifying the impacts of others on OS [104].

Although the brain is the organ consuming the most oxygen in the body, it is particularly vulnerable to OS due to its diminishing antioxidant defenses [104,106].

In the central nervous system, ROS is generated not only from oxidation but also from neurotransmitter degeneration [104]. Excess ROS results in lipid peroxidation, DNA damage, protein oxidation, neuroinflammation, neurodegeneration, reduced neuroplasticity, and impaired neurotransmitter production and function [104]. Elevated levels of oxidative biomarkers (e.g., malondialdehyde and 8-hydroxy-2′-deoxyguanosine) are consistently found in depressed individuals [111]. These changes in the central nervous system can cause symptoms of anxiety and depression [104]—an unsurprising finding, considering that brain regions like the hippocampus and prefrontal cortex, both of which are critical in MDD, are among the most affected by OS. Among proinflammatory molecules, IL-1β has the most potent effect size in relevance to trauma exposure, post-traumatic stress disorder, and depression [104,112,113,114]. Interestingly, repeated OS can initiate and maintain elevated levels of IL-1β, leading to a positive feedback loop between OS and neuroinflammatory [104].

Deeply understanding the feedback loop between psychological distress and OS opens the chance for potential intervention to break this cycle. Antioxidants and lifestyle modifications have the potential to improve the mental health of men and, by that, reduce the negative influences of stress on sexual dysfunction, particularly ED.

### 4.2. Smoking

Smoking is a widespread health-risk behavior that has long been known as a risk factor for a variety of NCDs, including CVDs, cancer, metabolic diseases, and chronic respiratory diseases [115]. There is no “risk-free” smoking: both active and passive cigarette smoking are considered risk factors for ED [116], and second-hand exposure to smoking is likewise hazardous to human health [117], particularly in cases of pre-pubertal exposure [118]. While behavioral, genetic, and environmental factors are also involved, the inhalation of significant amounts of chemical substances, such as nicotine, cotinine, NO, ROS, peroxynitrite, and free radicals originating from organic molecules is likely responsible for most of the systemic damage resulting from smoking; in particular, nicotine is not only one of the main factors affecting vascular response but also very psychologically addictive [119]. All these substances can promote systemic inflammation by activating inflammatory signaling pathways, which are the main drivers for several of the complications resulting from smoking, such as the development of cancer, lung fibrosis, and other health effects [120]. From a vascular point of view, these molecules stimulate ROS production within blood vessels, resulting in endothelial dysfunction, plaque progression, and thrombogenicity [23]. Penile vessels are particularly affected by smoking, as shown by several meta-analysis studies proving a significant risk of ED among smokers [121,122,123]. The use of heat-not-burn devices, while potentially safer than traditional tobacco smoking, is far from being risk-free [124]; however, it could be considered as part of more complex harm reduction techniques for people who are trying to quit smoking but are unable to [125], as suggested by the Italian Society of Andrology and Sexual Medicine [5].

### 4.3. Obesity

ED and obesity may share an internal pathologic environment, and the main pathophysiologic processes are OS, inflammation, and resultant insulin and leptin resistance [126].

Obesity affects over a third of the global population. In males, it explicitly impacts reproductive health, leading to ED, subclinical prostatitis, and reduced semen quality [127].

Obesity alters the hypothalamic–pituitary–gonadal (HPG) axis, leading to reduced testosterone levels and hypogonadism. It increases the aromatization of testosterone to estrogen in adipose tissue, further disrupting hormonal balance. Elevated levels of ROS in obese individuals damage sperm DNA, reduce motility, and impair chromatin condensation. Finally, obesity induces epigenetic changes, such as altered DNA methylation, that can negatively impact offspring health [128].

### 4.4. Environmental Contributors

OS is a central mechanism linking environmental exposure to hypogonadism and ED. ROS damages Leydig cells, disrupts the HPG axis, and interferes with NO bioavailability, a critical molecule for erectile function [129].

**Pesticides**: Several agents used in industrial agriculture can induce the formation of ROS, leading to lipid peroxidation, DNA damage, and disrupted testosterone synthesis in Leydig cells. Examples include diazinon, chlorpyrifos, and cypermethrin [130,131]. While the contaminants might be present in traces, continuous exposure can lead to bioaccumulation, which can result in more severe outcomes.

**Radiation (Ionizing and Non-ionizing)**: Exposure to different kinds of radiation can damage Leydig cells and testicular tissues via ROS-induced DNA damage and mitochondrial dysfunction. Sources include mobile phones, Wi-Fi, and medical radiotherapy [132,133].

**Air Pollution**: Heavy metals like cadmium (Cd) and lead (Pb) generate ROS, disrupting testosterone synthesis, damaging sperm, and inducing infertility [130,131]. Some of these pollutants are also byproducts of smoking [124,134].

## 5. OS and Non-Communicable Diseases

**Figure 3:**
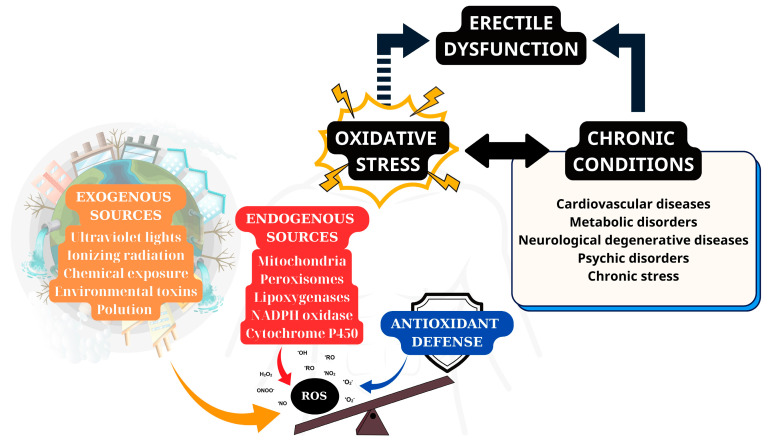
The vicious circle involving oxidative stress, erectile dysfunction, and chronic conditions.

There is a plethora of clinical conditions that prominently feature OS as either a causative factor or a consequence; however, in some cases, these conditions actually feature both. OS can, in fact, lead to the development of some conditions that would result in a decrease in the scavenging capabilities of the body or in further excess production of ROS [135,136,137]. In fact, from the perspective of sexual medicine, ED is an intermediate link in this vicious cycle of disease and is complexly affected by both OS and chronic health conditions (Figure 3). While this vicious circle requires careful management, both via lifestyle changes and (in some cases) medical intervention, to be adequately addressed, it could also be considered a therapeutic target [135].

The following conditions hereby described are particularly relevant for experts involved in andrology and medical sexology since they also affect sexual health, showing that interventions aimed at reducing OS can be effective at different levels to promote better sexual and systemic health.

### 5.1. Hypertension

Hypertension is one of the most common risk factors for ED, if not the most common [138]. OS and endothelial function are significantly altered in hypertensive individuals compared to normotensives [139,140,141], and OS has been suggested to be involved in the pathogenesis of hypertension and its complications [142]. More in detail, the importance of OS in hypertension lies in the critical role of ROS and redox signaling in molecular, cellular, and systemic processes [143]. These processes contribute to endothelial damage, vascular dysfunction, cardiovascular remodeling, renal impairment, sympathetic nervous system activation, immune cell stimulation, and systemic inflammation, all of which are pivotal in the pathophysiology of hypertension [143] and contribute to increasing the risks of cardiovascular morbidity and mortality [141,144]. Angiotensin II, a potent vasoconstrictor, plays a key role in the onset and persistence of hypertension. Its binding to the angiotensin I receptor in the arterial wall activates NADPH oxidase, resulting in the production of ROS [145]. NO is inactivated by ROS, reducing its bioavailability and impairing vascular relaxation. Increased ROS levels damage the endothelium, leading to vascular stiffness and impaired blood pressure regulation. ROS activates inflammatory pathways, which contribute to vascular remodeling and elevated blood pressure.

### 5.2. Diabetes Mellitus

Sexual dysfunctions are common in all patients with diabetes, particularly in type 2 diabetes mellitus (T2DM) patients with comorbid conditions such as obesity, hypogonadism, neurological involvement, or depressed mood [10]. With these patients, a more comprehensive strategy is needed [10]. In fact, research indicates a multitude of intertwining factors associated with diabetes and sexual dysfunctions—particularly ED, which has an extremely high prevalence in this population [24,146]. The cardiovascular risk profile in patients with diabetes is by itself a risk factor for the development and progression of ED [10], further increased by common comorbidities, particularly metabolic syndrome and dyslipidemia. Hyperglycemia, obesity, and hyperlipidemia all contribute to increased ROS production [147,148]. In diabetes, persistent hyperglycemia and mitochondrial dysfunction lead to elevated ROS production, intensifying OS [149]. Patients with T2DM and ED showed significantly increased circulating monocyte oxidative activity compared to those without ED [9]. Increased monocyte oxidative activity in ED patients points to subclinical endothelial damage, which is likely exacerbated by elevated LDL and OS. Last but not least, chronic low-grade inflammation is a common condition in T2DM, resulting from diabetes itself as well as from its comorbidities, which has an interdependent relationship with OS [150] and is involved in the pathogenesis of diabetes mellitus-associated ED [151].

### 5.3. Hyperlipidemia

Hyperlipidemia is a major risk factor for vasculogenic and neurogenic ED, with OS playing a key role in the onset of vasculogenic ED linked to hyperlipidemia [152]. Research shows that tissue from the corpora cavernosa of animals fed a high-cholesterol diet exhibits elevated ROS production [153,154]. In the penis, activated NAD(P)H oxidase serves as a primary source of OS, leading to eNOS uncoupling and contributing to endothelial dysfunction in hypercholesterolemia-induced ED [155].

### 5.4. Male Hypogonadism

Male hypogonadism is a clinical and biochemical syndrome featuring impaired testicular function, as expressed by reduced ability to produce testosterone and/or sperm cells [156,157,158,159,160]. Depending on its pathogenesis, male hypogonadism can be either primary (i.e., due to a testicular failure), secondary (i.e., due to a pituitary dysfunction), or tertiary (i.e., due to hypothalamic defects); however, a combination of factors is also possible, as occurring in late-onset hypogonadism [156,161]. Male hypogonadism has also been classified according to the reversibility of the condition as “organic” (i.e., non-reversible, due to a congenital or acquired permanent dysfunction of the HPG axis) vs. “functional” (i.e., reversible, associated with aging and various NCDs like obesity, diabetes, and CVD) [162]. Symptoms of male hypogonadism vary according to age, severity, and duration but generally include sexual dysfunction, reduced morning erections, fatigue, increased adiposity, decreased muscle and bone mass, and mood disturbances [156,157,158,159,160].

In hypogonadism, OS impacts steroidogenesis, primarily affecting Leydig cells and mitochondrial functions [163]. Increased ROS inhibit key steroidogenic enzymes, such as the steroidogenic acute regulatory (StAR) protein and cytochrome P450 [164], resulting in impaired testosterone production [165], which in turn, leads to impaired NO synthesis [166]. NO also reacts with ROS to form peroxynitrite, reducing NO bioavailability and contributing to vascular dysfunction and ED [167]. It is also worth mentioning that, in men, testosterone has an anti-inflammatory role [168], which might be suppressed in male hypogonadism, resulting in the development of an inflammatory status that can act as a potential contributing factor to the onset and progression of sexual dysfunctions. Therefore, OS can disrupt all mechanisms involved in erection by acting on neurogenic, vasculogenic, and endocrine factors often exacerbated by ROS-induced endothelial dysfunction.

### 5.5. Lower Urinary Tract Symptoms (LUTS)

Lower urinary tract symptoms (LUTS) are a heterogeneous complex of irritative or obstructive symptoms affecting the lower urinary tract in up to 70% of adults. Symptoms are categorized into storage (e.g., frequency, urgency), voiding (e.g., weak stream), and post-micturition issues (e.g., incomplete emptying) [169]. LUTS are common in both men and women; are linked to several conditions and NCDs, such as obesity, metabolic syndrome, diabetes, CVDs, and neurodegenerative disorders; and are increasingly prevalent with age [170]. LUTS are not inevitable with age but can be the manifestation of underlying pathologies. In men, the relationship between LUTS and ED has been well-established; thus, factors that can exacerbate LUTS also somewhat affect ED [171,172].

OS can be the bond between LUTS, aging, and chronic diseases [173]. In fact, OS can be a pathogenetic mechanism for several conditions (among which, undoubtedly, aging, obesity, and metabolic syndrome), which can be risk factors for the onset or progression of LUTS. However, OS can also directly be involved in the pathogenesis of urinary symptoms: there is, indeed, evidence that OS can promote bladder dysfunction, impair detrusor contraction, and result in bladder hyperactivity [169]. Similarly, the contractility of urethral sphincter muscles can be affected by ROS, resulting in muscle atrophy, collagen deposition, impaired urethral closure [174,175], and thus urethral dysfunction [176]. Unsurprisingly, the prostate is also heavily affected by OS. In fact, OS has been considered one of the main drivers for the development of benign prostatic hyperplasia (BPH) [177,178]. The exact mechanisms through which OS promotes the histological remodeling occurring in BPH have been extensively studied, and several mechanisms have been proposed, such as increased oxidative DNA damage, hyperactivation of the NF-κB pathway, and increased activity of enzymes involved in matrix remodeling, such as prolidase [169,179,180].

### 5.6. Gout

Gout is described as a chronic inflammatory condition with significant implications for quality of life, including sexual health [9], resulting from a persistent state of hyperuricemia, which leads to the deposition of monosodium urate crystals in target organs and tissues. Hyperuricemia is closely associated with the production of ROS since the enzyme xanthine oxidoreductase (XOR), which catalyzes the hydroxylation from hypoxanthine to xanthine to uric acid, produces ROS as a byproduct of the reaction. Uric acid has both antioxidant and pro-oxidant properties. However, in the pathogenesis of gout, it predominantly activates the immune response, contributing to the inflammatory cascade associated with gout flares [181] and with all the relevant systemic symptoms, including pain [182].

Gout is a systemic condition that can impair not only the physical abilities of affected patients but also their psychological health and quality of life due to reduced motility, poor hygiene, and feelings of inadequacy [183,184,185]. On top of the psychological burden of disease associated with gout, the inflammatory status exacerbates conditions like ED through endothelial dysfunction and reduced NO availability [40]. Sexual quality of life is impaired in men with gout [186], and poor clinical management of the disease can worsen sexual dysfunction due to systemic inflammation, OS, and psychosocial factors (“sexgout”) [187]. Treatment of gout is mainly focused on reducing serum uric acid levels and induction of crystal dissolution: to reach this objective, lifestyle changes and drugs acting on the XOR (allopurinol and febuxostat) should be used [182]. These strategies can also reduce the OS status of the affected patient, with beneficial effects for systemic health as well as for sexual function [9,187]. However, compliance to therapy is remarkably low among gout patients [188], and strategies to improve adherence to treatment are severely needed. Presenting improvements in sexual health as an “added benefit” to gout treatment might change the patients’ view in these regards and might also lead to a progressive involvement of the patient’s partner(s) in the clinical management of gout.

## 6. Other Sexual and Reproductive Dysfunctions

### 6.1. Premature Ejaculation

Premature ejaculation (PE) refers to ejaculation happening earlier than desired during sexual activity. It is the most prevalent sexual disorder in males, affecting approximately one-third of individuals [189]. PE and ED can coexist, with the two conditions often overlapping and influencing one another in one syndrome called Loss of Control over Erection and Ejaculation (LCEE) [190,191]. PE can either be lifelong, known as primary PE, or develop later in life, in which case it is classified as secondary PE. PE has several known risk factors, among which are endocrine disorders [192,193] and LUTS [169]; however, PE can be both a consequence or the cause of ED. In fact, PE and ED are closely interconnected, potentially creating a vicious cycle in which either sexual dysfunction could be a risk factor for the development of the other [194] or for the progression from subclinical to more severe forms [15,27]. Despite this mutual influence, the precise factors driving their relationship in the LCEE have only partially been addressed and warrant further investigation [195].

No direct evidence of involvement of OS in the pathogenesis of PE has been found so far; however, some connections can be drawn. First and foremost, as elsewhere stated, OS can act at different levels on the genitourinary tract, increasing the prevalence of conditions such as chronic prostatitis/chronic pelvic pain syndrome (CP/CPPS) and LUTS [169] that are recognized risk factors for PE [196]. Then, as shown by the present review, an imbalance between ROS production and antioxidant mechanisms can worsen erectile function, thus establishing the grounds for a decline in ejaculatory function as well. Recent evidence has suggested an anti-oxidant effect of dapoxetine [197], the main treatment for PE [198]; however, these data remain at present only available in experimental post-stroke animal models, and their clinical relevance deserves further investigation.

### 6.2. Male Infertility

Couple infertility is a public health issue, affecting as much as 15% of couples trying to conceive [199]. Infertile couples often complain of varying degrees of sexual disorders [200,201], and while an exact prevalence of these conditions has never been ascertained, studies report about a 20–60% rate of male sexual dysfunctions among infertile men [202]. Couple infertility can have a significant detrimental effect on the couple’s health and stability and can have a devastating psychological burden, which can often lead to clinical complications, among which, undoubtedly, sexual dysfunctions—to the point that the effects of infertility (and, by extension, of medically assisted reproduction) on sexual health have been defined as a full-fledged Inferto-Sex Syndrome [203]. However, it could also be argued that in some circumstances, sexual dysfunctions can be among the reasons for couple infertility: men who ejaculate before penetration or who are unable to perform sexual activity because of ED cannot achieve a natural conception.

When considering the association between ED and male infertility, OS is a risk factor that largely overlaps between the two conditions. In fact, OS is among the main hypothesized risk factors for male reproductive issues: while capacitation and acrosome reaction depend on physiologic OS, an imbalance between ROS production and impaired clearance can lead to OS within sperm [204]. This can subsequently affect the characteristics of the sperm: studies have indeed shown that OS can result in a higher frequency of sperm DNA damage, impaired motility, and altered membrane integrity [205,206,207,208,209]. OS occurs even within the sperm: while, in fact, spermatozoa have inherent scavenging capabilities, they are impaired in cases of teratozoospermia [210,211,212]. Based on these premises, antioxidant treatments have been extensively used to potentially improve sperm parameters and increase the likelihood of conception. However, the rationale for their use has often been questioned [213], and existing guidelines often suggest introducing antioxidant nutraceutical products only as a “last resort” following a complete diagnostic workup [214].

It could be argued that intervening in OS can be useful in the clinical management of infertile couples: in fact, as clearly shown in the present review, OS can have a negative effect on sexual health, reproductive function, and psychological status. The mutual relationship between the three is well-established, and as such, an approach aimed at reducing OS—a common risk factor for all three—can potentially improve clinical outcomes. However, rather than using anti-oxidant treatment, an adequate approach would involve acting on lifestyles [22], such as by reducing health-risk behaviors such as alcohol or smoking [134,215], by improving physical exercise [216], or when risk cannot be avoided, by enacting strategies aimed at harm reduction [125].

## 7. Conclusions: Reducing Oxidative Stress to Improve Erectile Function

Having established the vicious circle represented by the equation ED = ED, the role of Sexual Medicine (SM) in improving the NCDs studied by Systems Medicine (SM, so that another equation has been formulated: SM = SM) appears evident [24]. In fact, sexual health is a tremendously effective argument to induce patients to the most difficult task, i.e., modifying the lifestyle-related risk factors of NCDs, the major source of inflammation and oxidative stress. Also, for this reason, it is nonsensical to deal with NCDs without considering the patient’s and the couple’s sexual and relational health [217]. In the effort to modify their lifestyle, the couple, in fact, may have a dramatic impact. An impact that has been represented in the concept of the anti-inflammatory couple, as the dyadic relationship supporting reciprocally the need to quit smoking and abusing drugs, to reduce alcohol and calorie intake, to shift to better diet models, as the Mediterranean one, and to increase the daily physical exercise amount [187]. In fact, any strategy to reduce OS, represented by inflammation and hypoxia, reduces the risk of NCDs and, at the same time, sexual dysfunction, chiefly ED. At the same time, several treatments for ED may increase adherence to behavioral prescription, as those mentioned above, but may also have direct benefits in reducing OS. For example, one of the most effective and popular treatments for ED, the PDE5 inhibitor sildenafil, improves endothelial function in patients with a typical NCD such as diabetes [218,219]. In fact, since sildenafil and other PDE5 inhibitors can induce vasodilation in vascular beds other than the penile arteries, new therapeutic uses have been identified [220]. Last but not least, this class of drugs in general, and sildenafil in particular, now available in a convenient orodispersible film formulation [221,222,223], have been demonstrated to be very safe, if not protective, for use in patients suffering from oxidative stress such as those with CVDs [46], another reason to take care of sexual health.

## Figures and Tables

**Table 1:** All sources of reactive oxygen species (ROS). NO = nitric oxide; ED = erectile dysfunction; SOD = superoxide dismutase.

Category	Source	Description	Example/Notes
Endogenous Sources	Mitochondria	Electron leakage during oxidative phosphorylation generates superoxide.	Major contributor to oxidative damage in tissues; linked to ED via reduced NO availability and endothelial dysfunction.
	NADPH Oxidase (NOX)	Transmembrane enzymes transfer electrons from NADPH to O_2_, producing superoxide.	Critical for immune defense (e.g., neutrophils), but excess ROS causes tissue damage (e.g., hypertension, diabetes).
	Xanthine Oxidase (XO)	Catalyzes hypoxanthine → xanthine → uric acid, releasing ROS.	Elevated in liver damage, hypoxia, or inflammation; unclear role in ED.
	Uncoupled NOS	NOS isoforms (eNOS, nNOS, iNOS) produce superoxide instead of NO when uncoupled.	eNOS uncoupling in penile tissue contributes to ED and local oxidative stress.
Exogenous Sources	Environmental Pollutants	Heavy metals (Fe, Cu, Cd, Hg), industrial solvents, air pollution.	Trigger ROS via redox cycling or direct oxidation.
	Lifestyle Factors	Smoking, alcohol, UV/X-ray radiation, poor diet.	Directly induce ROS production or impair antioxidant defenses.
	Drugs	Halothane, doxorubicin, and metronidazole	Generate ROS as a side effect, exacerbating oxidative stress.
Degenerated Antioxidant Defenses	Dysfunctional Antioxidant Enzymes	SOD, catalase, glutathione peroxidase lose activity due to mutations or damage.	Failure to neutralize ROS leads to accumulation (e.g., SOD dysfunction in aging).
	Autoxidation of Antioxidants	Reduced glutathione (GSH) or vitamin C reacts with transition metals (Fe, Cu).	Paradoxically generate ROS (e.g., Fenton reaction).
	Degradation of Antioxidants	Polyphenols, carotenoids, or flavonoids degrade into pro-oxidants.	Prolonged heat/light exposure converts antioxidants into ROS-promoting compounds.
	Antioxidant Imbalance	Excess antioxidants react with ROS to form unstable intermediates.	“Antioxidant paradox”: High antioxidant levels may exacerbate oxidative stress.

## Data Availability

No data to be shared.

## Abbreviations

The following abbreviations are used in this manuscript:

ED	Erectile dysfunction
CVD	Cardiovascular disease
NCD	Non-Communicable Disease
LCEE	Loss of control of erection and ejaculation
NO	Nitric oxide
NOS	Nitric oxide synthase
nNOS	Neuronal nitric oxide synthase
eNOS	Endothelial nitric oxide synthase
PDE5	Phosphodiesterase Type 5
OS	Oxidative stress
ROS	Reactive oxygen species
RNS	Reactive nitrogen species
ONOOH	Peroxynitrous acid
ONOO–	Peroxynitrite
ICAM-1	Intercellular Adhesion Molecule 1
VCAM-1	Vascular Cell Adhesion Molecule 1
NOX	NADPH oxidase
XO	Xanthine oxidase
iNOS	Inducible nitric oxide synthase
SOD	Superoxide dismutase
GSH	Glutathione
MAPK	Mitogen-activated protein kinases
ERK1/2	Extracellular signal-regulated kinases 1 and 2
MDD	Major depressive disorder
IL-1	Interleukin 1
IL-6	Interleukin 6
TNF	Tumor Necrosis Factor
HPG	Hypothalamic–pituitary–gonadal
T2DM	Type 2 diabetes mellitus
LUTS	Lower urinary tract symptoms
BPH	Benign Prostate Hyperplasia
XOR	Xanthine oxidoreductase
PE	Premature ejaculation
CP/CPPS	Chronic prostatitis/chronic pelvic pain syndrome
